# Emergence of hypomania and mania following initiation of a ketogenic diet: case series

**DOI:** 10.3389/fnut.2026.1815787

**Published:** 2026-04-08

**Authors:** Christopher M. Palmer

**Affiliations:** Department of Psychiatry, Metabolic and Mental Health Program, McLean Hospital, Harvard Medical School, Belmont, MA, United States

**Keywords:** bipolar disorder, hypomania, ketogenic diet, mania, metabolic psychiatry

## Abstract

The ketogenic diet is a century-old, evidence-based treatment for drug-refractory epilepsy, and is now also utilized as an evidence-based treatment for obesity and type 2 diabetes. Emerging research suggests that it may play a role in the treatment of serious mental illness, including treatment-resistant depression, bipolar disorder, and schizophrenia. As with any powerful therapeutic intervention, there can sometimes be side effects. This case series of 9 participants describes 8 who experienced hypomania and 1 who experienced mania within 2 months of starting a ketogenic diet. Of note, 7 of the participants did not have a prior history of a bipolar-spectrum disorder. None of the participants required hospitalization or the addition of mood-stabilizing medications as a result of these mood episodes. Nonetheless, individuals and clinicians should be aware of this risk when using the ketogenic diet, monitor for symptoms of hypomania and mania, and use appropriate strategies to manage symptoms if they emerge. These findings also raise intriguing questions and offer possible insights into the phenomenology of hypomania and mania.

## Introduction

The ketogenic diet is a fasting-mimicking diet that is low in carbohydrates, high in fat, and moderate in protein. It was developed over 100 years ago as a treatment for epilepsy and is now recognized as an evidence-based treatment for drug-resistant epilepsy (1). It can stop seizures even when medications fail to do so. There are many variations of the ketogenic diet, including ones that require strict measurement of foods and macronutrients. The diet can consist primarily of animal-sourced or plant-sourced foods or a combination of the two. Epilepsy treatments are routinely used in the treatment of psychiatric disorders, so it is not surprising that the ketogenic diet is gaining interest as a treatment for serious mental illnesses, including treatment-resistant depression, bipolar disorder, schizophrenia, and other psychiatric disorders.

The first reported use of the ketogenic diet as a treatment for serious mental illness was in 1965 when researchers placed 10 hospitalized women with schizophrenia on a ketogenic diet and reported improvement in symptoms within 2 weeks (2). Over the past decade, interest in the ketogenic diet as a treatment for psychiatric disorders has increased significantly. At least 5 preclinical studies of murine models of schizophrenia and several case reports have been published demonstrating the efficacy of this intervention as a possible treatment for schizophrenia (3). A 2024 comprehensive review identified 53 publications representing almost 2,000 individuals, indicating that the ketogenic diet can reduce symptoms of a wide range of psychiatric diagnoses, including schizophrenia, bipolar disorder, depression, Alzheimer’s disease, and autism-spectrum disorder (4). A systematic review and meta-analysis of ketogenic diets compared to control interventions found a significant antidepressant effect, although an anti-anxiety effect was uncertain (5). Additional randomized controlled trials of the ketogenic diet as a treatment for a wide range of mental disorders are now underway.

Numerous mechanisms of action have been proposed to explain how and why the ketogenic diet might improve symptoms of serious mental illness. These include improvements in glucose hypometabolism, neurotransmitter activity, oxidative stress, and inflammation (6). Additionally, mitochondrial dysfunction may play a key role in these disorders and can be improved with ketogenic diet therapy (7).

Given the growing interest in the ketogenic diet as a treatment for mental disorders, as well as its increasing use as a treatment for obesity and type 2 diabetes (8), it is important to be aware of any adverse effects or risks associated with the diet.

This case series describes 9 participants who developed hypomania or mania within 2 months of initiating a ketogenic diet. 7 of the participants did not have a prior history of a bipolar-spectrum disorder.

## Case series description

The 9 participants in this case series represent either clinical or community cases identified by the author who reported hypomanic or manic symptoms after starting a ketogenic diet. All participants were interviewed to assess age, gender, reason for trying the diet, weight at the time of initiation of the ketogenic diet, assessment of the presence of ketones, total weight loss from the ketogenic diet when episodes began, past and current psychiatric history, a retrospective and current assessment using the Young Mania Rating Scale (YMRS), the duration of hypomanic or manic symptoms, and any interventions that were used to reduce or manage the hypomanic or manic symptoms. The YMRS is a validated and frequently used instrument for the assessment of mania and hypomania (9). It includes 11 items, and scores can range from 0–60. While there are no formally validated scores that define hypomania or mania, a score of 12 is sometimes considered the minimum threshold for hypomania.

The participants included 5 males and 4 females with an average age of 44 years (range: 31–59 years). At the time of starting the ketogenic diet, 3 participants met criteria for major depressive disorder, with 1 of them also meeting criteria for co-morbid borderline personality disorder; 1 met criteria for bipolar II disorder; 1 met criteria for schizoaffective disorder, bipolar type; 2 had a history of major depressive disorder; and the remaining 2 had no current or past psychiatric history. See Table 1 for demographic information and psychiatric diagnoses for each participant.

**Table 1:** Demographic information and psychiatric diagnoses of participants.

Participant number	Gender	Age in years (reported as a range to de-identify participants)	Current psychiatric diagnoses and duration	Past psychiatric diagnoses	Number of days on diet before symptoms began	Weight loss (lbs) when symptoms began	Duration of hypomanic/Manic episode (days)	Peak YMRS score
1	M	47–52	None	Major depressive disorder, OCD	35	30	165	16
2	M	53–58	Major depressive disorder (2 years)	Major depressive disorder	60	21	42	24
3	F	46–51	None	None	29	11	30	23
4	M	23–28	Bipolar II disorder (11 years)	Bipolar II	3	2	6	24
5	M	31–36	Schizoaffective disorder, bipolar type (8 years)	Schizoaffective disorder, bipolar type; OCD; substance use disorder	10	15	14	32
6	F	54–59	Major depressive disorder (1 year)	Major depressive disorder	20	8	14	15
7	M	46–51	None	None	21	22	180	19
8	F	34–39	Major depressive disorder (8 years) Borderline personality disorder (15 years)	Major depressive disorder, borderline personality disorder	14	5	14	13
9	F	43–48	None	None	18	17	112	12

## Diagnostic assessment

Participants experienced the onset of hypomanic or manic symptoms within 3 to 60 days of initiating the ketogenic diet (median of 20 days). In 8 of the participants, symptoms began within the first month of starting the diet. Only the subject with preexisting schizoaffective disorder was classified as mania due to concurrent psychotic symptoms. The remaining participants all met criteria for a hypomanic episode. None of them required hospitalization due to the manic or hypomanic symptoms.

All participants verified that they were in ketosis either through urine acetoacetate testing (7 participants) and/or blood measurement of beta-hydroxybutyrate (4 participants), although specific values of blood and urine ketone measurements were highly variable. All participants lost weight, an average of 14 pounds (range: 2–30 pounds), before symptoms of hypomania or mania began.

**Figure 1:**
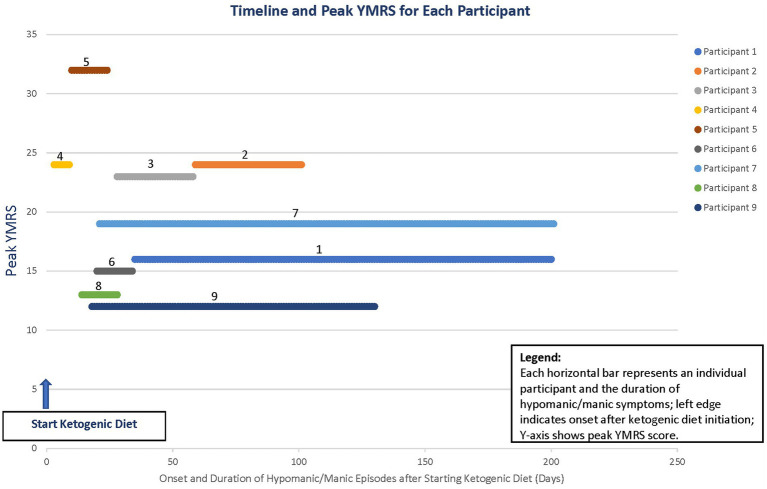
Timeline of the onset and duration of hypomanic and manic symptoms.

Prior to starting the diet, all participants had YMRS scores between 0 and 2, except for the participant with preexisting schizoaffective disorder, who had a score of 10 due to pre-existing psychotic symptoms. At the peak of hypomanic or manic symptoms, the average YMRS score was 19 (range: 12–32). Symptom duration ranged from 6 to 180 days, with an average of 67 days (Figure 1). The predominant symptoms were elevated mood, decreased need for sleep (with some participants sleeping only 2 h per night for several days), and increased activity. Irritability and disruptive behaviors occurred only in the participant with preexisting schizoaffective disorder.

In response to the symptoms of hypomania or mania, 3 participants stopped the ketogenic diet on their own due to concerns about the lack of sleep, 1 participant increased carbohydrate intake by about 15 g per day while remaining in ketosis, and 2 took sleeping medication for up to 2 weeks to restore normal sleep. At the time of the final interview, 6 of the 9 participants remained on the ketogenic diet because they were experiencing benefits of either weight loss and/or improvement in mental symptoms. All symptoms of hypomania and mania resolved in all participants back to baseline YMRS scores without the use of mood stabilizing or additional antipsychotic medications.

## Discussion

This is the first published case series of the ketogenic diet being associated with the onset of hypomania, particularly in people without preexisting bipolar-spectrum disorder. While this case series identifies a possible risk associated with the ketogenic diet, it also raises important questions about the underlying nature and treatment of bipolar disorder.

In patients with preexisting bipolar disorder, there have been prior reports in the medical literature that fasting and the ketogenic diet may be associated with mood episodes. One study followed 20 Muslim bipolar patients through the month of Ramadan, during which they fasted from sunrise to sunset. 45% of the patients experienced a mood episode during Ramadan, with 71% of those episodes being the manic phase of the illness (10). Even the patients who did not have full mood episodes experienced increased insomnia and anxiety, both associated with increased risk of a mood episode. In 2005, there was a case report of the Atkins diet, which is often ketogenic, causing mania with psychotic features in a 54-year-old man with bipolar disorder (11). He was hospitalized, taken off the diet, and his symptoms resolved.

There have been many studies that have assessed the effects of fasting, intermittent fasting, or calorie restriction on mood, fatigue, sleep, and other symptoms of mood disorders. For example, one study recruited 34 young, healthy men and women and instructed them to fast from dawn to dusk for 30 days in order to simulate Ramadan (12). They found a decrease in fatigue and depression scores in these participants, suggesting an antidepressant effect in participants who were not clinically depressed at the time. This study, however, did not report any symptoms of hypomania or mania in the participants.

There are additional, albeit sparse, case reports in the medical literature of fasting causing symptoms consistent with hypomania, although none of the authors identified the symptoms as hypomanic symptoms. For example, a study published in 1959 described 9 obese patients who were hospitalized and fasted for 4–9 days (13). The author noted that “…many reported a marked sense of well-being, suggesting a mild euphoria,” and, “Several patients were given a mild sedative at night for sleep…” Another study published in 1966 hospitalized 13 obese patients and fasted them for 25–249 days (14). The authors noted, “all our patients spontaneously commented on their increased sense of well-being, and in some, this amounted to frank euphoria.” Neither study suggested that these patients required further psychiatric intervention, even though both are suggestive of hypomanic or manic symptoms in response to fasting.

One of the strengths of this case series is that all patients were assessed using a standardized rating scale for mania and hypomania, and all of the participants confirmed through objective tests that they were in ketosis. Some weaknesses include some of these assessments being conducted retrospectively, and the 9 participants differing from each other in terms of current and past psychiatric diagnoses. Additionally, it’s not clear how often or frequently manic or hypomanic symptoms might emerge in response to a ketogenic diet, as this series did not include participants on the ketogenic diet who did not experience these symptoms. Nonetheless, this case series and the existing medical literature report instances of hypomanic or manic symptoms occurring in some people following the initiation of a ketogenic diet or fasting. This is important information for individuals, clinicians, and researchers who are using or studying the ketogenic diet, particularly in patients with preexisting bipolar disorder.

This case series raises important and intriguing questions about the etiology of bipolar-spectrum disorders. The observation that hypomanic and manic symptoms have occurred in some people following fasting or a fasting-mimicking diet could lead to better insights into the origins of these mood states. Extensive literature highlights the roles of neurotransmitter function, inflammation, oxidative stress, mitochondrial function, and insulin signaling in bipolar disorder, all of which can be influenced by the ketogenic diet (15). Additionally, sleep disruption may be another relevant mechanism. One meta-analysis of studies looking at sleep metrics in people participating in Ramadan diurnal intermittent fasting found that, on average, total sleep time in participants decreased from 7.2 h to 6.4 h per night during Ramadan (16). Since sleep deprivation is known to trigger hypomanic and manic episodes in susceptible individuals, this may be a contributing factor.

This case series raises the question of whether hypomania might be a normal and adaptive response to a fasting state. Although the first days of fasting are usually associated with hunger, fatigue, and irritability in most people, it is somewhat surprising that longer fasting episodes can produce hypomania, even if only in some people. One can imagine situations of food scarcity in which enhanced energy, motivation, confidence, and decreased need for sleep might help an animal acquire food successfully. Neural circuits that warn of starvation may play a role in the activation of hypomanic symptoms in order to motivate the animal to explore, fight enemies, forage, or hunt in order to get food. Given that starvation is a clear threat to the animal, this hypomanic state might confer an adaptive advantage for survival. All of the participants in this case series lost weight prior to experiencing hypomanic or manic symptoms. It’s not clear if weight loss, ketosis, or a combination of the two is associated with the emergence of symptoms. Future research could begin to explore these distinctions.

This case series also raises important diagnostic and management questions regarding bipolar-spectrum disorders. Most mental disorders are syndromes defined by clusters of symptoms and have multifactorial, incompletely understood etiologies. Many symptoms of mental disorders, such as depression and anxiety, are normal, adaptive traits in the right environmental circumstances. The field is less clear on whether symptoms of hypomania can also be normal and adaptive in the right circumstances. Given that 7 of the participants had no prior history of a bipolar-spectrum disorder, it raises the question of whether to diagnose a bipolar-spectrum disorder in this context. Three of the participants met criteria for major depression when they started the diet, and 2 had a prior history of major depression. According to the Diagnostic and Statistical Manual of Mental Disorders: DSM-5-TR, they should now be diagnosed with bipolar II disorder (17). Treatment guidelines for bipolar II disorder generally recommend lifetime maintenance treatment with mood stabilizers (18). So, based on the emergence of hypomania in these 5 participants, their lifetime diagnoses and psychiatric management could be dramatically impacted. Nonetheless, none of these participants started mood stabilizing medications, and all of their symptoms of hypomania resolved within 180 days, with an average duration of 67 days. Two of the participants did not have a history of mood episodes, so they do not meet criteria for bipolar II disorder. The question of whether to diagnose them with a bipolar-spectrum disorder is unclear and inconsistent in DSM-5-TR. There is a diagnosis of “Other Specified Bipolar and Related Disorder (F31.89).” Criteria for this disorder can include a hypomanic episode without a prior major depressive episode. However, the overall criteria for this disorder state that the disorder must “cause clinically significant distress or impairment in social, occupational, or other important areas of functioning.” However, DSM-5-TR defines a hypomanic episode as “… not severe enough to cause marked impairment in social or occupational functioning or to necessitate hospitalization.” Neither of these participants experienced marked impairment in social or occupational functioning, so they were not diagnosed with a psychiatric disorder.

In thinking about the use of the ketogenic diet as a treatment for mental disorders, some might be alarmed at the risk of hypomania and mania. It is important to point out that most biological treatments in psychiatry have been associated with the emergence of mania and hypomania in at least some people. This includes antidepressant medications (19), antipsychotic and mood stabilizing medications (20–22), electroconvulsive therapy (23), repetitive transcranial magnetic stimulation (24), transcranial direct current stimulation (25), and even bright light therapy (26). All of these are evidence-based treatments for a variety of psychiatric disorders, and we continue to use them despite this risk. If anything, the fact that the ketogenic diet might induce hypomania or mania in some people lends further support to its impact on the brain and its possible use as a treatment for psychiatric disorders.

Patients, clinicians, and researchers should be aware of the possible risk of hypomania or mania when initiating a ketogenic diet, monitor for symptoms, and use appropriate strategies to manage episodes if they emerge. Future prospective studies are needed to determine the incidence, risk factors, and clinical significance of these observations. Additional research on fasting, the ketogenic diet, and their effects on mood states may help to further elucidate the etiology of bipolar and other mood disorders and aid in the development of more effective treatment strategies.

## Data Availability

The original contributions presented in the study are included in the article/supplementary material, further inquiries can be directed to the corresponding author.
